# Dual Specificity Phosphatase 5 Is Essential for T Cell Survival

**DOI:** 10.1371/journal.pone.0167246

**Published:** 2016-12-09

**Authors:** Raman G. Kutty, Gang Xin, David M. Schauder, Stephanie M. Cossette, Michelle Bordas, Weiguo Cui, Ramani Ramchandran

**Affiliations:** 1 Developmental Vascular Biology Program, Division of Neonatology, Department of Pediatrics, Department of Obstetrics and Gynecology, Children’s Research Institute, Medical College of Wisconsin, Milwaukee, Wisconsin, United States of America; 2 Department of Cell Biology, Neurobiology, and Anatomy, Medical College of Wisconsin, Milwaukee, Wisconsin, United States of America; 3 Blood Research Institute, BloodCenter of Wisconsin, 8727 Watertown Plank Road, Milwaukee, Wisconsin, United States of America; 4 Department of Microbiology and Molecular Genetics, Medical College of Wisconsin, Milwaukee, Wisconsin, United States of America; University of Alabama at Birmingham, UNITED STATES

## Abstract

The mitogen-activated protein kinase (MAPK) pathway regulates many key cellular processes such as differentiation, apoptosis, and survival. The final proteins in this pathway, ERK1/2, are regulated by dual specificity phosphatase 5 (DUSP5). DUSP5 is a nuclear, inducible phosphatase with high affinity and fidelity for ERK1/2. By regulating the final step in the MAPK signaling cascade, DUSP5 exerts strong regulatory control over a central cellular pathway. Like other DUSPs, DUSP5 plays an important role in immune function. In this study, we have utilized new knockout mouse reagents to explore its function further. We demonstrate that global loss of DUSP5 does not result in any gross phenotypic changes. However, loss of DUSP5 affects memory/effector CD8^+^ T cell populations in response to acute viral infection. Specifically, *Dusp5*^*-/-*^ mice have decreased proportions of short-lived effector cells (SLECs) and increased proportions of memory precursor effector cells (MPECs) in response to infection. Further, we show that this phenotype is T cell intrinsic; a bone marrow chimera model restricting loss of DUSP5 to the CD8^+^ T cell compartment displays a similar phenotype. *Dusp5*^*-/-*^ T cells also display increased proliferation, increased apoptosis, and altered metabolic profiles, suggesting that DUSP5 is a pro-survival protein in T cells.

## Introduction

In response to infection, naïve T cells circulating in the periphery recognize their cognate antigen and undergo activation. These activated T cells differentiate into either short-lived effector cells (SLEC) or memory precursor effector cells (MPEC). SLECs are highly cytotoxic but have low memory potential while MPECs have decreased cytotoxic capabilities and increased memory potential. These MPECs eventually develop into mature memory T cells [1]. As a result of their differentiation, SLECs have a high apoptotic potential and lose the ability to self-renew, whereas MPECs have low apoptotic potential and readily self-renew. Upon reinfection, mature memory cells rapidly differentiate into SLEC and MPEC cells, providing both faster and more efficient clearance of pathogen. Both cell types are readily defined by their surface protein expression of two key proteins: killer cell lectin-like receptor subfamily G member 1 (KLRG1) and CD127. CD127, also known as interleukin-7 receptor alpha (IL-7Ra), is one unit of the heterodimer interleukin 7 (IL-7) receptor. KLRG1 is a surface marker with unknown function, but serves to differentiate SLEC and MPECs. Specifically, SLECs have high KLRG1 expression and low CD127 expression. MPECs up-regulate CD127 and lose KRLG1 expression. Therefore, SLECs and MPECs are also termed KLRG1^+^/CD127^-^ and KLRG1^-^/CD127^+^ cells, respectively.

Our laboratory has been studying regulators of the MAPK pathway, in particular the dual-specificity phosphatases (DUSPs). We study the fifth member of this family, DUSP5, which is a nuclear phosphatase protein whose expression is induced by cytokines, stress, and other stimulatory factors. DUSP5 dephosphorylates residues T202/T185 and Y204/Y187 of pERK1/2, respectively, leading to ERK1/2’s inactivation [2]. DUSP5 regulates ERK1/2 with high affinity and fidelity, and ERK1/2 are the only known substrates of DUSP5 [3–6]. Additionally, DUSP5 has been reported to be an important mediator of immune function and is expressed in T cells [7]. DUSP5 was first reported to be induced by interleukin 2 (IL-2) and has since been shown to be induced by a host of interleukins including IL-7, IL-12, IL-15, and, more recently, IL-33 [3, 8, 9]. In addition to T cells, DUSP5 is induced or highly expressed in B cells, eosinophils, dendritic cells, macrophages, and mast cells [10, 11]. Other studies have examined the role of DUSP5 *in vivo* using mouse models [9, 12–14]. These papers show a clear function for DUSP5 in the immune system in addition to other tissues. However, no studies as of yet have established its role in CD8^+^ T cells following infection. Given that DUSP5 expression is strongly induced in T cells by stress and interleukin signaling and DUSP5 has been shown to regulate cellular survival in eosinophils, we hypothesized that DUSP5 is critical for T cell survival in a stressed host environment. In this study we investigated the role of DUSP5 in T cell survival following infection.

## Materials and Methods

### Mice

All mouse experiments were performed under the approved Medical College of Wisconsin IACUC Animal Protocol AUA1022. Animals used in this study were group housed in a 12-hour light/12-hour dark cycle with free access to food (standard mouse chow) and water (chlorinated water). For additional enrichment, animals were also provided Enviro-Dri nesting material. Animals were monitored by lab staff and animal facility staff, which included full-time veterinarians. Humane endpoint determination was assessed using a scoring system that included the following criteria: body weight change, physical appearance, respiratory rate, and behavioral response to external stimuli. If an animal scored 3 or higher in any category or received a cumulative score of 9 or higher it was considered to be in distress and was humanly euthanized. No mice met the criteria for humane endpoints during the length of these studies. For tissue collection and completion of the mouse studies the mice were humanely euthanized via CO_2_ inhalation followed by cervical dislocation.

**Fig 1:**
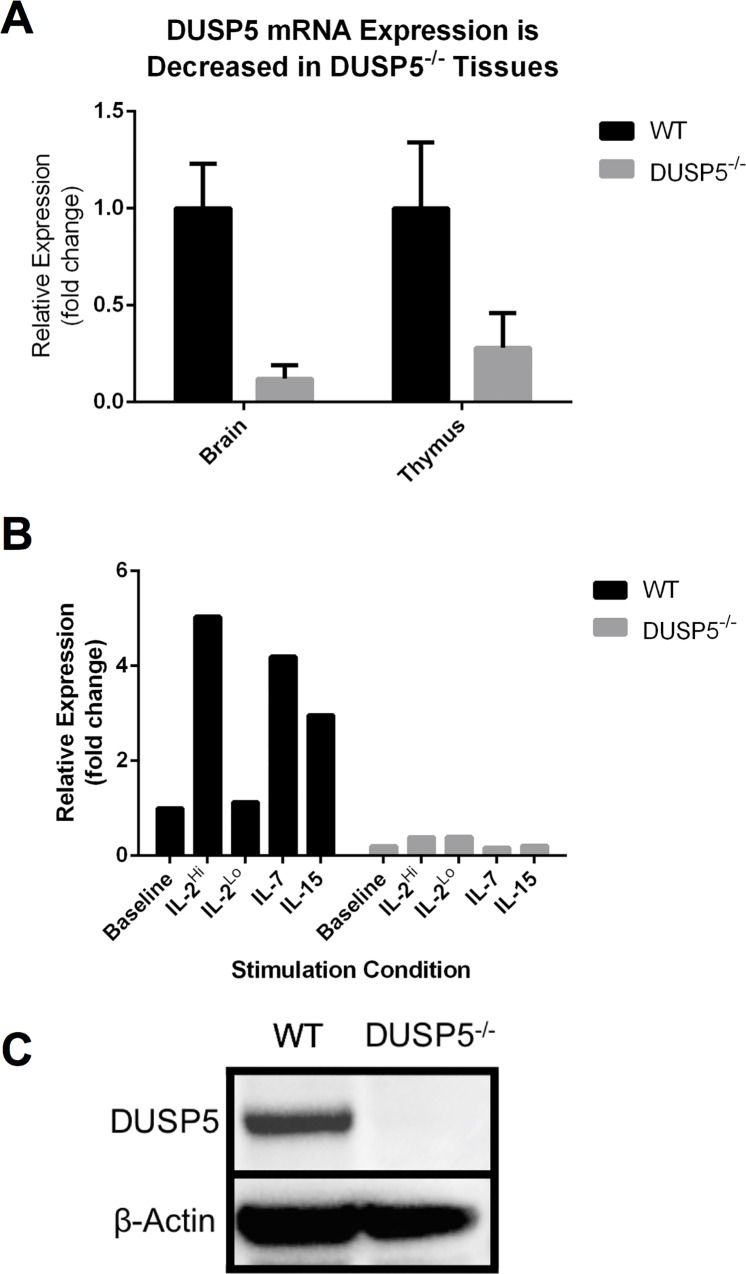
*Dusp5*^*-/-*^ mice do not express DUSP5. A: DUSP5 mRNA expression was measured by qPCR in thymus and brain tissues in WT and *Dusp5*^*-/-*^ adult mice. B: Similarly, DUSP5 mRNA expression was measured in WT and *Dusp5*^*-/-*^ T cells cultured with several interleukins known to induce DUSP5 expression. C: DUSP5 protein expression was measured in WT and *Dusp5*^*-/-*^ SLEC T cells cultured in IL-2.

Mice heterozygous for the *Dusp5* “Knockout first” (-) targeting allele (Dusp5^tm1a(KOMP)Wtsi^, mutant cell line EPD0575_3_B05; S1A Fig) were obtained from The Wellcome Trust Sanger Institute Mouse Genetics Project (Sanger MGP) [15]. In this construct the *Dusp5* gene is interrupted with a Neomycin (*neo*) cassette as well as *lacZ* reporter cassette. Translated products are terminated before exon 2 via an early stop codon at the end of the *neo* cassette (S1A Fig) [16]. This large insertion results in a protein-null allele (Fig 1C). Mice were genotyped using the following primers: WT-Forward 5’-AGGCTAGGGGAAAAGCCAGTTATGG-3’, WT-Reverse 5’- GAGAGACCAGAGAGACCAATCCTGC-3’, *Dusp5*^*-/-*^ -Forward 5’- TCTTATCATGTCTGGATCCGGGG-3’ (WT 609bp, *Dusp5*^*-/-*^ 350bp). Mice were backcrossed to the C57BL/6 line for a minimum of 5 generations (N5) prior to collection of experimental data. *Dusp5*^*-/-*^ mice were crossed with Actin beta-driven FLP recombinase mice (B6.Cg-Tg(ACTFLPe)9205Dym/J mice, Jackson Laboratory, Bar Harbor, Maine) in order to excise the lacZ and neomycin cassettes. Progeny were crossed with CMV-driven Cre recombinase mice (B6.C-Tg(CMV-cre)1Cgn/J mice, Jackson Laboratory) to excise (exc) the second exon of *Dusp5*, thus generating mice with the *Dusp5*^-/-(Exc)^ allele. Littermates were then crossed to generate *Dusp5*^*-/-(Exc)*^ mice (S6A Fig).

### Quantitative Polymerase Chain Reaction (qPCR)

Tissue samples and cells were collected from WT and *Dusp5*^*-/-*^ mice and RNA was extracted using TRIzol reagent (ThermoFisher, Waltham, MA) and standard protocol. Total cDNA was made from 1μg RNA per sample using SuperScript III reverse transcriptase with random primers (ThermoFisher). 18S and *Dusp5* were amplified with the following primers: *18S* forward: 5’-GTAACCCGTTGAACCCCATT-3’; 18S reverse: 5’-CCATCCAATCGGTAGTAGCG-3’; *Dusp5* forward: 5’-GAAAGCCCGTTCTCAGCGT-3’; *Dusp5* reverse: 5’-CGAGGAACTCGCACTTGGAT-3’. qPCR reaction was performed using EvaGreen 2x qPCR MasterMix (Midwest Scientific, Valley Park, MO). Samples were normalized to 18S levels and then *Dusp5*^*-/-*^ samples were normalized to WT.

### Lymphocytic Choriomeningitis Virus (LCMV) Infection

Adult *Dusp5*^+/+^ and *Dusp5*^*-/-*^ mice were infected with intraperitoneal injection of 2x10^5^ pfu LCMV Armstrong in PBS. Blood was drawn at days 8, 15, 30, and 40 to assess viral progression and T cell compartment expansion and contraction (S2 Fig). During the first week of infection the mice were monitored daily for the expected signs of general malaise, minor weight loss, reduced activity level, ruffled fur and a hunched appearance. These symptoms typically resolved one-week post-infection. Although no animals in this study required the following treatments, animals that did not display signs of improvement would have been provided with extra soft bedding and supplemented with fluids until the symptoms were no longer observed. Mice were then monitored weekly until the endpoint of 40 days. All mice were sacrificed 40 days post-infection via CO_2_ inhalation followed by cervical dislocation.

### Generation of Bone Marrow Chimeric Mice

10-week old (C57BL/6 CD45.1^+^) *Dusp5*^*+/+*^ mice were purchased from Charles River. Mice were injected with anti-CD8 antibody and then, after 1 week, lethally irradiated (900cGy with ^137^Cs γ-irradiator). Bone marrow (BM) from *CD8*^-/-^, *Dusp5*^*+/+*^, and *Dusp5*^*-/-*^ mice were isolated and mixed in a 7:3 ratio of CD8^-/-^ BM to either *Dusp5*^+/+^ or *Dusp5*^*-/-*^ BM in PBS supplemented with ampicillin (S3 Fig). Mice were anesthetized with isoflurane and then injected retro-orbitally with approximately 1x10^6^ BM cells. Animals were given antibiotic supplemented water (400mg/mL sulfadiazine/trimethoprim) for 2 weeks post-irradiation to prevent infection. Bone marrow reconstitution was assessed after 6 weeks via flow cytometry analysis of staining for CD45.1 and CD45.2. Normal mice require, at minimum, 6 weeks to reconstitute. Thus, mice were given a 6-week rest period before reconstitution was confirmed. A minimum of 25% CD45.2^+^ CD8^+^ T cells was required for a mouse to be considered sufficiently reconstituted.

### Restimulation

Splenocytes were isolated from LCMV-infected mice upon sacrifice on day 40. 2x10^6^ cells from each mouse were plated into 96-well plates, with one row for stimulation and another row for unstimulated controls. Cells were stimulated with GP33 antigen and incubated at 37°C for 6 hrs. After this incubation period, cells were stained for surface and intracellular proteins and then analyzed using flow cytometry.

### Flow Cytometry

All flow experiments were performed on either the LSRII or LSRII Special Order flow cytometers at the Flow Core of the Blood Research Institute. Antibodies used are listed in S1 Table.

### Cell Culture

Spleens and inguinal lymph nodes of C57BL/6 WT and *Dusp5*^*-/-*^ mice were isolated and processed into single-cell suspensions on 70μm cell strainers. Naïve CD8^+^ T cells were isolated first by removal of red blood cells with red blood cell lysis buffer (Sigma, 11814389001) followed by magnetic purification (EasySep Mouse Naïve CD8^+^ T cell kit, StemCell Technologies, 19858). Cells were seeded at 1x10^6^ cells per well in a 24-well plate. Cells were cultured in CD8 T cell media (RPMI-1640 supplemented with 1mM sodium pyruvate, 10mM HEPES buffer, 1x Pen/Strep, 292μg/mL L-glutamine, 1x MEM amino acids, 50μM ß_2_-mercaptoethanol and 10% FBS) supplemented with soluble anti-CD28, plate-bound anti-CD3, and low-dose IL-2 (1ng/mL, Peptech). Cells were activated in these conditions for 3 days after which they were sub-cultured in either SLEC growth conditions (T cell media supplemented with 10ng/mL IL-2) or MPEC growth conditions (T cell media supplemented with 10ng/mL IL-15). After 3 days of differentiation, cells were harvested for further applications (S4 Fig).

### Western Blot

Spleen and lymph node tissues were isolated from *Dusp5*^*-/-*^ and C57BL/6 WT mice as described above. CD8^+^ CD44^-^ naïve T cells were purified from these tissues, then activated and differentiated. Cells were differentiated in high IL-2 SLEC media as DUSP5 is difficult to detect in unstimulated tissues and IL-2 is the strongest inducer of DUSP5 expression. Whole cell lysates were isolated in Radioimmunoprecipitation assay (RIPA) buffer and 25μg of protein was separated on a 4–20% gradient SDS-PAGE gel. After transfer to PVDF membranes, the blots were blocked in 5% bovine serum albumin (BSA) in Tween-20 tris-buffered saline (TBST) (0.1% Tween-20 in TBS) for 1 hour and incubated overnight at 4°C with monoclonal antibody against DUSP5 (1:1000; Abcam, AB200708) and for 1 hour at room temperature with monoclonal antibody against β-actin (1:10,000; Sigma, A5441). HRP-conjugated mouse-anti-sheep and anti-rabbit secondary antibodies were used at 1:10,000 dilutions. Blots were incubated with a 1:100 mixture of Femto:Pico substrates (SuperSignal West Femto, 34095, Thermo Scientific, SuperSignal West Pico, 34078, Thermo Scientific) and exposed using a ChemiDoc Touch imaging system (Bio-Rad, Hercules, CA).

### Proliferation and Apoptosis Assays

T cells were cultured as described above. Samples were taken at days 4 and 6 to assess both proliferation and apoptosis. For proliferation data, 1x10^5^ cells were fixed and stored in 70% ethanol. Fixed cells were stained using FxCycle PI/RNase Staining Solution (Product F10797, ThermoFisher, Waltham, MA) according to protocol. Samples were run on a BD Accuri C6 cytometer and analyzed using FCS Express. For apoptosis data, 1x10^5^ cells were stained using the Annexin V Apoptosis Detection Kit (Product 556547, BD Pharmingen, San Diego CA) according to protocol. Samples were run on an LSRII flow cytometer and analyzed using FlowJo (FlowJo LLC, Ashland, OR).

### Metabolism Studies

Splenic-derived T cells were cultured as described above. On day 6, live T cells were harvested and plated in a 24-well SeahorseXF plate at a seeding density of 5x10^5^ cells per well in basal RPMI media supplemented with either IL-2 or IL-15, 25mM glucose, and 1mM pyruvate. T cells were subjected to mitochondrial stress tests as described previously [17]. Oxygen consumption rate (OCR) was normalized to the time point immediately before the injection of oligomycin. This point additionally functioned as the basal respiratory rate. Maximal OCR was defined as the highest measured OCR after the addition of FCCP but before the addition of Antimycin A. The spare respiratory capacity (SRC), then, was the difference between the maximal OCR and the basal rate. *Dusp5*^*-/-*^ SRC was further normalized to WT SRC for fold change comparison.

### Statistics

All data were presented as mean value ± SEM. Data were analyzed using two-tailed T tests with p-values <0.05 considered statistically significant. For expected breeding genotype distribution data, variables were compared using a chi-square analysis. All statistical analysis was performed using Prism software (GraphPad Software, La Jolla, CA).

## Results

### Generation and Characterization of the *Dusp5*^*-/-*^ Mice

*Dusp5*^*-/-*^ mice are born in expected Mendelian ratios (S1C Fig), survive to adulthood, and show no apparent gross phenotype–all consistent with other reports [9, 13, 14], and indicative that DUSP5 is dispensable for embryonic development. Previous studies showed elevated expression of *Dusp5* in brain and thymic tissues [18]. Therefore, we isolated RNA from both WT and *Dusp5*^*-/-*^ mice and performed qPCR, demonstrating loss of *Dusp5* in both tissues (Fig 1A). *Dusp5* is consistently yet lowly expressed in lymphoid tissues, particularly T cells [10, 18]. However, upon stimulation with interleukins -2, -7, and -15, DUSP5 is dramatically induced in WT T cells, and nearly undetected in *Dusp5*^*-/-*^ cells (Fig 1B). Given that IL-2 induces the strongest expression of *Dusp5*, we cultured cells in high-IL-2 and probed the lysates for DUSP5. We were unable to detect DUSP5 in *Dusp5*^*-/-*^ lysates (Fig 1C). These results collectively indicate *Dusp5* is not expressed in our animal model.

### Examination of T cell Populations in *Dusp5*^*-/-*^ Mice During Acute Infection

**Fig 2:**
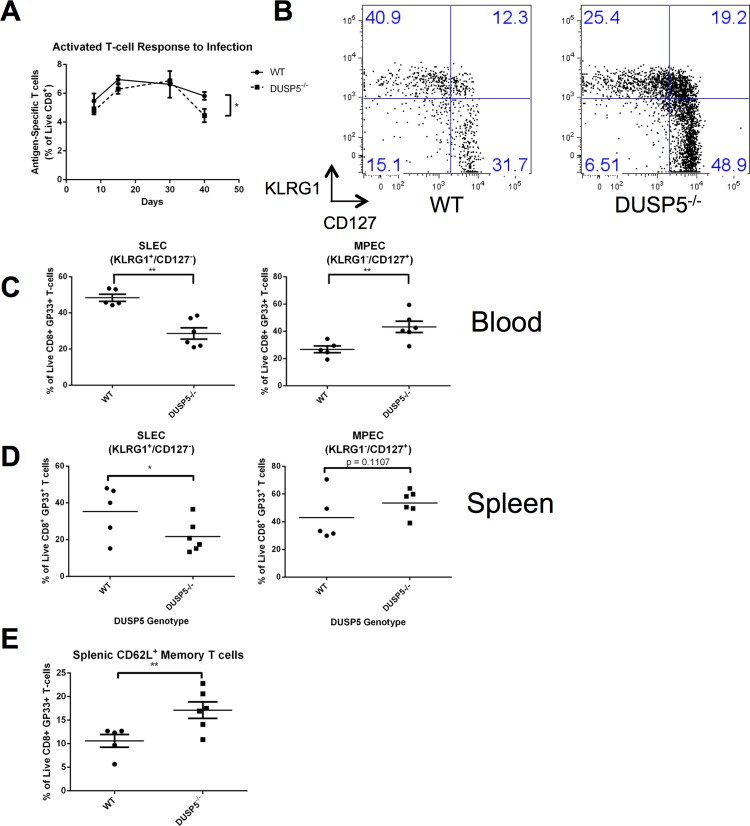
Global loss of DUSP5 alters effector/memory CD8^+^ T cell balance *in vivo*. A: Time course tracking LCMV-specific CD8^+^ T cells during the 40-day time course. B: Comparison of CD8^+^ SLECs (KLRG1^+^/CD127^-^) and MPECs (KLRG1^-^/CD127^+^) in blood in WT and *Dusp5*^*-/-*^ mice. C: Comparison of CD8^+^ SLECs (KLRG1^+^/CD127^-^) and MPECs (KLRG1^-^/CD127^+^) in spleen in WT and *Dusp5*^*-/-*^ mice. D: Analysis of total memory cells (CD62L^+^) in spleens of WT and *Dusp5*^*-/-*^ mice. n = 5 for WT and n = 6 for *Dusp5*^*-/-*^. *: p<0.05, **: p<0.01 ***: p<0.005.

A recent study has shown that knockout animals do not have alterations in basal T cell populations during homeostasis [9]. Indeed, in this same study eosinophil populations were unchanged until challenged with parasitic infection. Kovanen et al described alterations in T cell populations; however this was in a DUSP5 transgenic mouse [12]. Considering the present knowledge regarding DUSP5’s role in the immune system and its induction in stress, we subjected WT and *Dusp5*^*-/-*^ mice to acute lymphocytic choriomeningitis virus (LCMV) to determine if DUSP5 impacted T cell response to infection. LCMV-Armstrong is a well-characterized viral strain that elicits a strong lymphocytic response. Further, robust reagents are available that allow for tracking of responding T cells. Briefly, WT and *Dusp5*^*-/-*^ mice were infected with LCMV Armstrong and blood samples were taken 8, 15, 30, and 40 days after infection (Fig 2A). We observed differences in SLEC and MPEC populations on day 40 via cellular staining for KLRG1 and CD127 (Fig 2B). We found that the proportion of SLECs, identified as KLRG1^+^/CD127^-^, was significantly decreased 40 days after infection and the proportion of MPECs, identified as, KLRG1^-^/CD127^+^ was significantly increased 40 days after infection in both blood (Fig 2C) and in spleen (Fig 2D) in 4-month old *Dusp5*^*-/-*^ mice compared to age-matched WT mice. Furthermore, memory CD8^+^ cell frequency was increased in *Dusp5*^*-/-*^ mice (Fig 2E). While we observed these changes in both the blood and spleen, we did not observe any population changes in the bone marrow or lymph nodes of infected mice (data not shown). Therefore, DUSP5 is required for T cell survival in response to acute infection, and more specifically, promotes SLEC cell survival.

### DUSP5 Functions Intrinsically in T cell Development

**Fig 3:**
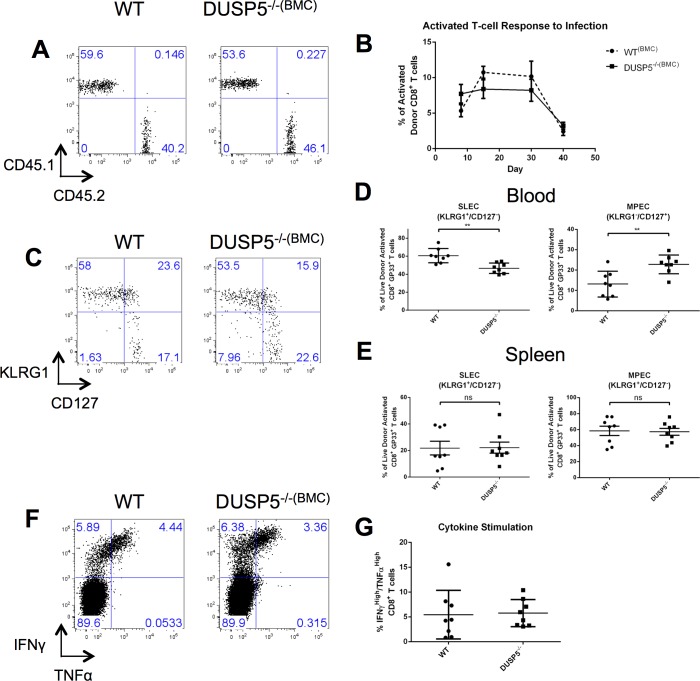
DUSP5 regulates T cell populations intrinsically in the blood but extrinsically in the spleen. A: Bone marrow chimeras expresses donor (CD45.2^+^) CD8^+^ T cells. B: Time course tracking LCMV-specific CD8^+^ T cells during the 40-day time course. C: Representative plot showing KLRG1 vs CD127 expression of CD8^+^ GP33^+^ activated T cells. D: Comparison of CD8^+^ SLECs (KLRG1^+^/CD127^-^) and MPECs (KLRG1^-^/CD127^+^) in blood in WT and *Dusp5*^*-/-(BMC)*^ mice. E: Comparison of CD8^+^ SLECs (KLRG1^+^/CD127^-^) and MPECs (KLRG1^-^/CD127^+^) in spleen in WT and *Dusp5*^*-/-(BMC)*^ mice. F: Representative plot showing IFNγ vs. TNFα expression in stimulated CD8^+^ splenocytes. G: Comparison of frequencies of IFNγ^+^/TNFα^+^ double positive CD8^+^ T cells. Together, these suggest a CD8^+^ T cell-intrinsic role for DUSP5 in regulating CD8^+^ T cell populations in the blood in response to infection and a CD8^+^ T cell-extrinsic role for DUSP5 in other tissues. n = 8 for both WT and *Dusp5*^*-/-(BMC)*^. *: p<0.05, **: p<0.01 ***: p<0.005.

While our initial studies did show alterations in CD8^+^ T cell survival following acute infection, the use of global knockouts did not provide sufficient resolution to determine if the phenotype is T cell intrinsic or extrinsic. To address this question, bone marrow chimera (BMC) mice were created such that DUSP5 deficiency was restricted to the CD8^+^ T cell compartment (denoted *Dusp5*^*-/-(BMC)*^) while the majority of other immune cells were WT (S3 Fig). Importantly, donor mice express CD45 isoform 2 (CD45.2) while host mice express CD45.1. This allowed us to track host vs. donor T cells reliably. Flow cytometry data taken before infection showed robust proliferation of donor (CD45.2) T cells (Fig 3A). These mice were infected as described above and their respective CD8^+^ T cell populations were tracked throughout the infection period using flow cytometry analysis (Fig 3B). We observed similar results (decreased SLECs, increased MPECs) in the blood of *Dusp5*^*-/-(BMC)*^ animals compared to WT^BMC^ animals (Fig 3C and 3D). Surprisingly, we did not observe these alterations in SLEC/MPEC CD8^+^ T cell populations in spleens isolated from these animals (Fig 3E). We additionally examined these T cells for effector function by measuring their ability to produce cytokines in response to antigen. Splenic T cells were stimulated with cognate antigen as described above. Unstimulated controls and stimulated T cells were stained for intracellular IFNγ and TNFα. We found no statistical difference between WT^(BMC)^ and *Dusp5*^*-/-(BMC)*^ T cells in their ability to produce IFNγ^+^/TNFα^+^ effector T cells (Fig 3F and 3G). Collectively, these findings show that DUSP5 has CD8^+^ T cell-intrinsic functions, particularly in regulating survival of SLECs and MPECs. Furthermore, DUSP5 does not influence effector function in these T cells.

### DUSP5 is Required Intrinsically for T cell Metabolism and Survival

**Fig 4:**
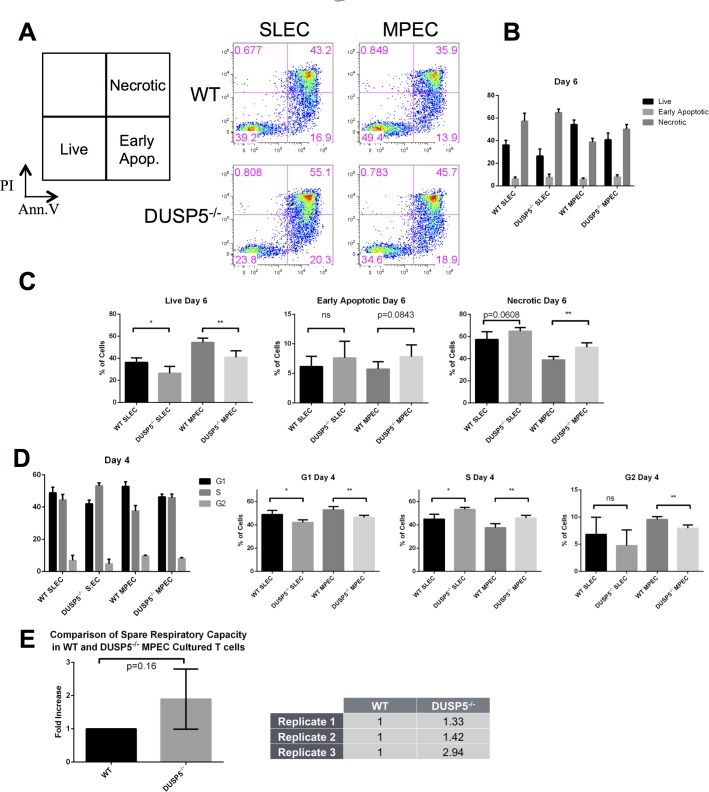
T cell survival is regulated by DUSP5. A: Legend for Annexin V/ Propidium Iodide flow plots (left) and representative flow plots of WT and *Dusp5*^*-/-*^ samples (right). B: Overall comparison of apoptotic populations in T cells after 6 days culture *in vitro*. C: Individual comparison of live cell, early apoptosis, and necrotic cell populations. *Dusp5*^*-/-*^ CD8^+^ T cells show significant reductions in live cells in both SLEC and MPEC conditions and significant increases in necrotic cells in MPEC culture conditions. D: Overall comparison of cell cycle populations in T cells after 4 days culture *in vitro* and specific comparison of G1, S, and G2 populations. *Dusp5*^*-/-*^ T cells show increases in proportion of cells in S phase and decreases in proportions in G1 and G2 phases in both SLEC and MPEC cultured T cells. E: *Dusp5*^*-/-*^ T cells have increased spare respiratory capacity (SRC) compared to WT T cells. SRC is a key survival mechanism in memory T cells. Together, these data show that DUSP5 plays an important role in T cell survival. Plots are means with error bars ±SEM. Comparisons were made using a two-tailed t-test, *: p<0.05, **: p<0.01 ***: p<0.005.

MAPK is a central regulator of cellular survival, proliferation, and metabolism. In order to explain our observations that MPECs increased in frequency *in vivo*, we sought to determine if this change was caused by decreased apoptosis, increased proliferation, or altered metabolic flux. For apoptosis, we stained splenic-derived, cultured T cells with AnnexinV (AV) and propidium iodide (PI) to discern live (AV^-^/PI^-^), early apoptotic (AV^+^/PI^-^), and necrotic cells (AV^+^/PI^+^) (Fig 4A and 4B). On day 6 of culture, both *Dusp5*^*-/-*^ SLEC and MPEC T cells show significant reductions in live cells and significant increases in necrotic cells in MPEC culture conditions (Fig 4C). Intriguingly, no changes were observed immediately after T cell receptor activation (data not shown) or after 1 day of interleukin stimulation (S5 Fig). These data imply that DUSP5 enacts its pro-apoptotic regulation of T cells not through T cell receptor (TCR) signaling and activation but instead after, when cells have more fully differentiated. Further, these data were confirmed in the *Dusp5*^*-/-(Exc)*^ mouse line, demonstrating that these results are due to the loss of *Dusp5* and not additional genetic modifications (S6B Fig).

In addition, we examined the proliferation of WT and *Dusp5*^*-/-*^ cultured cells using DNA propidium iodide (PI) staining. *Dusp5*^*-/-*^ cells demonstrated significantly decreased fraction of cells in G1 and G2 phase and a significant increase in proportion of cells in S phase on day 4 (Fig 4D). We observed these alterations in proliferation phase populations in both SLECs and MPECs. We did not observe any changes in proliferation at day 6 (data not shown). These data suggest an anti-proliferative role for DUSP5 in the G1/S phase checkpoint in early differentiation of T cells.

Another critical mechanism for T cell survival is metabolism. Studies have shown that effector cells rely more heavily on glycolysis while memory T cells rely on fatty acid oxidation [19–21]. Additionally, memory T cells have proportionately larger numbers of mitochondria and thus have a higher spare respiratory capacity (SRC) than other T cells [22]. It is theorized that this high potential for metabolism affords memory T cells the immense energy necessary to rapidly proliferate in response to re-introduction of antigen [23]. Considering the defects we observed in T cell survival both *in vivo* and *in vitro*, we assessed T cells for alterations in their baseline metabolism. We observed that *Dusp5*^*-/-*^ cultured MPECs had a demonstrably higher SRC than their WT counterparts (Fig 4E) and therefore, increased mitochondrial function. We observed no changes in metabolic stress response in cultured SLECs and no changes in either cell type in terms of glycolytic stress response (data not shown). These data show that not only does DUSP5 have a pro-apoptotic role in both SLECs and MPECs, but it also has a pro-survival role in MPECs.

## Discussion

In this study, we provide evidence for DUSP5 as a regulator of T cell survival. To evaluate DUSP5’s role in T cells, we created global knockout mice, subjected them to acute infection, and monitored their T cell profile for 40 days. We observed in the absence of DUSP5 a decreased frequency of short-lived effector cells (SLECs) with an increased frequency of memory precursor effector cells (MPECs) following acute infection compared to their WT cohorts in both the blood and spleen. These findings suggest that DUSP5 is required for the maintenance of SLECs *in vivo*. However, the global knockout model cannot determine if the phenotype is T cell intrinsic or extrinsic. To address this issue, we created bone marrow chimera (BMC) mice, which allowed for distinction between intrinsic and extrinsic roles of DUSP5 specifically in the CD8^+^ T cell compartment. Intriguingly, these BMC mice showed the same phenotype as the global knockouts in T cells in the blood (decreased SLECs and increased MPECs in *Dusp5*^*-/*-*(BMC)*^). However, we were not able to recapitulate the global *Dusp5*^*-/-*^ phenotype in the spleen. This suggests that DUSP5 alters SLEC survival in a tissue-specific context. It is possible that another cell type in the spleen is responsible for maintaining T cell populations and does so through the control of DUSP5, likely CD4^+^ T cells. Endothelial cells in the spleen are another candidate because our previous work demonstrated a role for DUSP5 in this cell type [24].

Additionally, we asked whether the loss of DUSP5 would result in changes in the ability of CD8^+^ T cell effector function. From a gross perspective, *Dusp5*^*-/-(BMC)*^ mice appeared as healthy as their WT counterparts on day 40, indicating that they were able to properly clear infection. Still, given the wide array of interleukins stimulating DUSP5, its control of a central pathway, and its reported function in other immune cell types, we hypothesized that DUSP5 also regulates T cell function. To investigate this hypothesis, we collected T cells from infected spleens at day 40 and stimulated them in vitro with GP33 antigen for effector function. We defined effector function by the production of IFNγ and TNFα. When stimulated with antigen we observed no differences in either the number of these cells or the mean fluorescence intensity (MFI) of either marker when comparing *Dusp5*^*-/-(BMC)*^ mice to WT^(BMC)^ mice. This suggests that while T cell population numbers are altered, these cells retain similar effector function to WT animals. Further, this indicates that DUSP5 is not responsible for regulating CD8^+^ T cell function.

Collectively, our data suggests a model wherein DUSP5 regulation of T cell survival *in vivo* leads to differences in SLEC and MPEC populations when challenged with acute infection. From both the global knockout data and the BMC data this seems most likely. However, an alternative model cannot be excluded, which is that DUSP5 is responsible for T cell proliferation instead. We reasoned that the loss of DUSP5 would enhance cell proliferation, resulting in alterations in T cell populations. We showed that loss of *Dusp5*^*-/-*^ in T cells *in vitro* increases proliferation in SLECs and MPECs on day 4, but these observed changes disappear on day 6. This falls in line with previous studies; the G1/S checkpoint is regulated by p53, which in turn regulates DUSP5 expression [25]. It is likely that DUSP5 acts as a negative feedback regulator on p53; loss of DUSP5 releases the p53 brake on T cell proliferation. This results in premature advancement of T cells through the G1/S checkpoint early in differentiation. Given the transient nature of this phenotype, it is unlikely that proliferation is responsible for the significant changes in MPEC/SLEC populations *in vivo*. From these results it is most likely that DUSP5 is acting as a regulator of T cell survival and not T cell proliferation.

Populations of specific cell types are generally maintained by proliferation and apoptosis, with proliferation increasing cellular number and apoptosis decreasing it. Both of these mechanisms are under the control of MAPK signaling. Given the observed changes in cellular proliferation, we also assessed the role of DUSP5 in T cell survival by examining those cells’ apoptotic rates *in vitro*. Using AnnexinV and propidium iodide staining, we showed that *Dusp5*^*-/-*^ T cells do in fact have higher rates of apoptosis. Intriguingly, this difference was only observed at day 6 of culture and not day 4. These data align well with the cell-survival hypothesis suggested earlier from the BMC data, and will warrant further exploration. Also, there is support in the literature for DUSP5 as a regulator of cell survival [9].

From these data, the role of DUSP5 as a regulator of T cell survival becomes clear. Extrapolating from the *in vitro* data, DUSP5 functions in early differentiation (day 4 *in vitro*) to temper proliferation and in later differentiation (day 6 *in vitro*) to attenuate apoptosis. While the relation to cell survival through reducing apoptosis is clear, regulation of proliferation is not immediately clear. While proliferation is necessary for the survival of cells, proliferation also robs cells of energy that is critically needed for other processes. By carefully regulating the rate of proliferation early on, DUSP5 ensures the energetic stability of these cells and therefore, their survival. Similarly, cells that prematurely advance into the cell cycle are more likely to undergo apoptosis. Given the high apoptotic potential for SLECs *in vivo* it is likely that they may be more affected by this phenomenon, driving SLEC populations down in the *Dusp5*^*-/-*^ infection studies. It is also possible that these two events are not related but instead point to two distinct temporally regulated functions for DUSP5 in the cell. However, we prefer the hypothesis that DUSP5 regulates the concerted mechanisms of cellular survival.

Numerous studies have connected survival pathways with mitochondrial function in immune cells. For example, BCL-X_L_ (pro-survival protein) regulates mitochondrial survival by regulating and stabilizing the inner membrane potential [26]. DUSP5 was discovered to control BCL-X_L_ expression in eosinophils and therefore regulates their survival as well [9]. Further, recent studies implicate mitochondrial function and metabolism as critical regulators of T cell differentiation [27]. An important survival mechanism for memory T cells is their reliance on oxidative phosphorylation, which can be measured through oxygen consumption and, more specifically, spare respiratory capacity (SRC). Studies have shown that memory cells increase their SRC, presumably to meet high metabolic demands upon re-introduction of antigen. Although not statistically significant, we observed a reliable increase in SRC in *Dusp5*^*-/-*^ cultured MPECs. This increased capacity might be a compensatory mechanism for the decreased survival resulting from loss of DUSP5. Our apoptosis and metabolism data together suggest that T cells are caught between regulatory pathways that control cell survival and functional effects. Recently, it was reported that the biphenolic compound honokiol activates mitochondrial SIRT3, a sirtuin deacetylase family protein, which is critical for mitochondrial function in mice. Importantly, this honokiol-mediated activation reversed cardiac hypertrophy [28]. Histone deacetylase inhibitors and DUSP5 participate in an integral regulatory signaling circuit that controls cardiac hypertrophy [29].

In summary, we have identified a novel function for DUSP5 in CD8^+^ T cells in differentiating and maintaining SLEC and MPEC populations. Although it is clear that DUSP5 is essential for T cell survival, additional work is necessary to unravel the underlying mechanisms associated with DUSP5’s pro-apoptotic and anti-proliferative effects in T cells.

## Supporting Information

S1 Fig A: genomic map of DUSP5 knockout-first allele indicating position of *lacZ* and *neo* cassettes. Exon 2 is floxed for tissue-specific excision of DUSP5. B: agarose gel for genotyping *Dusp5*^*-/-*^ mice. Lower band (350bp) represents DUSP5^-^ allele and upper band (609bp) represents WT allele. C: Chi-Square analysis of DUSP5 genotype distribution. No significant difference (p = 0.62) was detected between observed genotypes and expected genotypes. n = 210.
(TIF)

S2 Fig Schematic of LCMV infection model. WT and *Dusp5*^*-/-*^ mice are infected at day 0 with blood samples collected at days 8, 15, 30, and 40. At 40 days animals were sacrificed and organs were harvested for T cell population analysis via flow cytometry.
(TIF)

S3 Fig Schematic for generation of bone marrow chimeras. WT Ly5.1 (CD45.1) mice were lethally irradiated and subsequently injected with a mixture of *Cd8*^*-/-*^ bone marrow and either *Dusp* WT or *Dusp5*^*-/-*^ bone marrow in a ratio of 70:30. This was done to ensure that while *Dusp5* was not expressed in CD8^+^ T cells, other lymphoid cell types would have *Dusp5* expression. Once bone marrow was sufficiently reconstituted, mice participated in the LCMV infection model as described in S2 Fig.
(TIF)

S4 Fig In vitro cell culture model. Spleen and lymph node were isolated from mice and reduced to single-cell suspension. These suspensions were purified for CD8^+^ CD44^-^ naïve T cells and activated with anti-CD3 and anti-CD28 antibodies for three days. Cells were then sub-cultured into SLECs via IL-2 supplemented media or MPECs via IL-15 supplemented media. After 3 days of subculture, cells were collected for *in vitro* experiments.
(TIF)

S5 Fig T cells show no alterations in cell survival at day 4 of cell culture. Neither SLEC nor MPEC cultured cells showed any differences between live, early apoptotic, or necrotic cells. Cell viability was decided using AnnexinV/Propidium Iodide staining and flow analysis.
(TIF)

S6 Fig To ensure if the *in vitro* and *in vivo* data are due to elimination of DUSP5 protein expression and not due to other genetic alterations (either the neomycin or lacZ cassettes) *Dusp5*^*-/-*^ mice were crossed to excise these cassettes. A: schematic of crossing strategies to first remove the lacZ/neo cassettes and, second, to remove the second exon of DUSP5 (this line then termed “*Dusp5*^*-/-(Exc)*^”. Lane description in gel image: Ladder (L), Dusp5^-/-(exc)^ (1,2) and DUSP5^WT/WT^ (3). WT alleles produced 1500bp PCR products while Dusp5^-/-(exc)^ alleles produced 661bp PCR products. B: confirmation of phenotype. T cells from *Dusp5*^*-/-(Exc)*^ mice were isolated and cultured as described above, with apoptosis data collected as also described. For each sample, n = 3, *: p<0.05, **: p<0.01 ***: p<0.005, ****p<0.001.
(TIF)

S1 Table List of all flow antibodies used in this study. (TIF)
