# Supplementary figures and images for: Dual Specificity Phosphatase 5 Is Essential for T Cell Survival
Source: PLoS One. 2016 Dec 9;11(12):e0167246. doi: 10.1371/journal.pone.0167246 (PMC5147890; doi:10.1371/journal.pone.0167246)

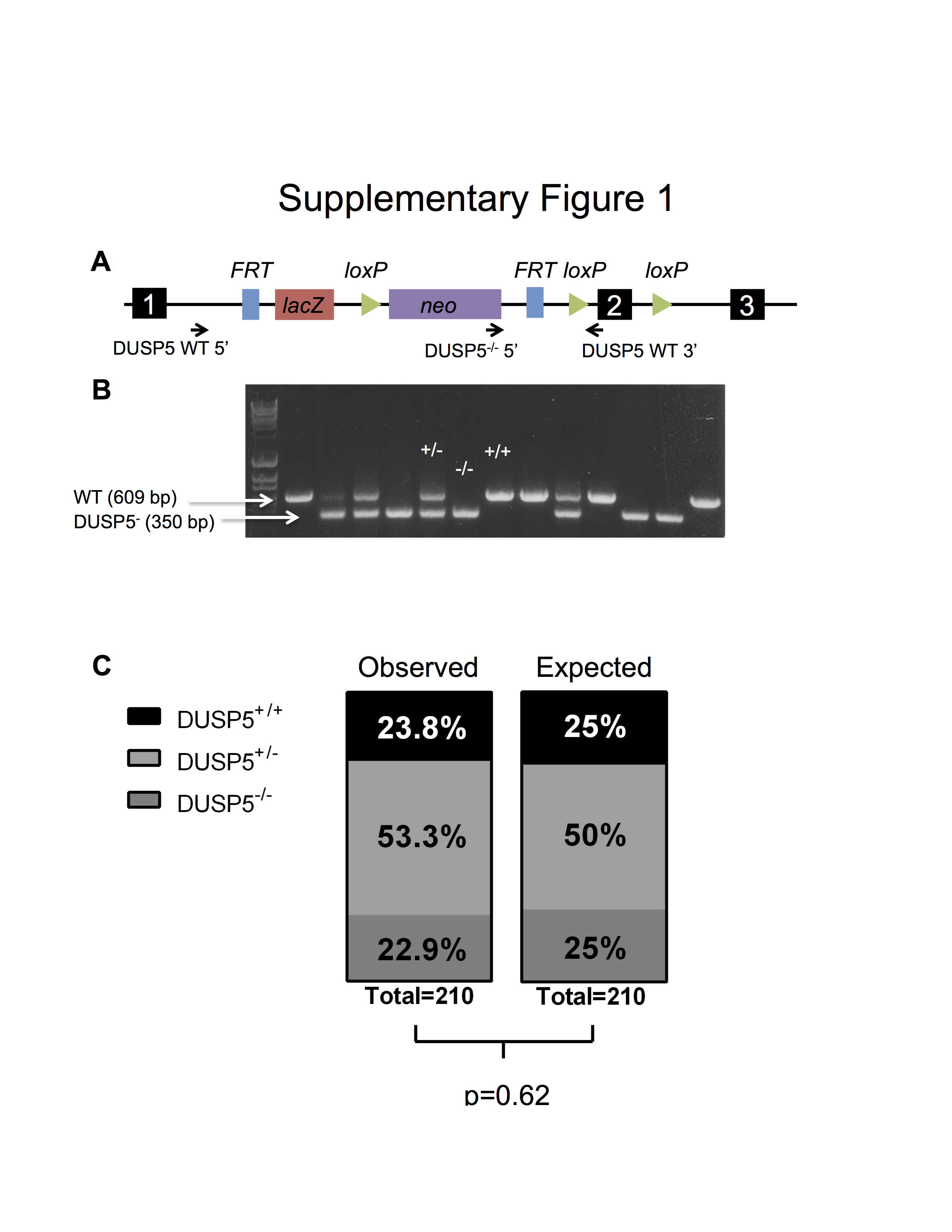

Supplement: S1 Fig — A: genomic map of DUSP5 knockout-first allele indicating position of lacZ and neo cassettes. Exon 2 is floxed for tissue-specific excision of DUSP5. B: agarose gel for genotyping Dusp5-/- mice. Lower band (350bp) represents DUSP5- allele and upper band (609bp) represents WT allele. C: Chi-Square analysis of DUSP5 genotype distribution. No significant difference (p = 0.62) was detected between observed genotypes and expected genotypes. n = 210. (TIF) [file pone.0167246.s001.tif]

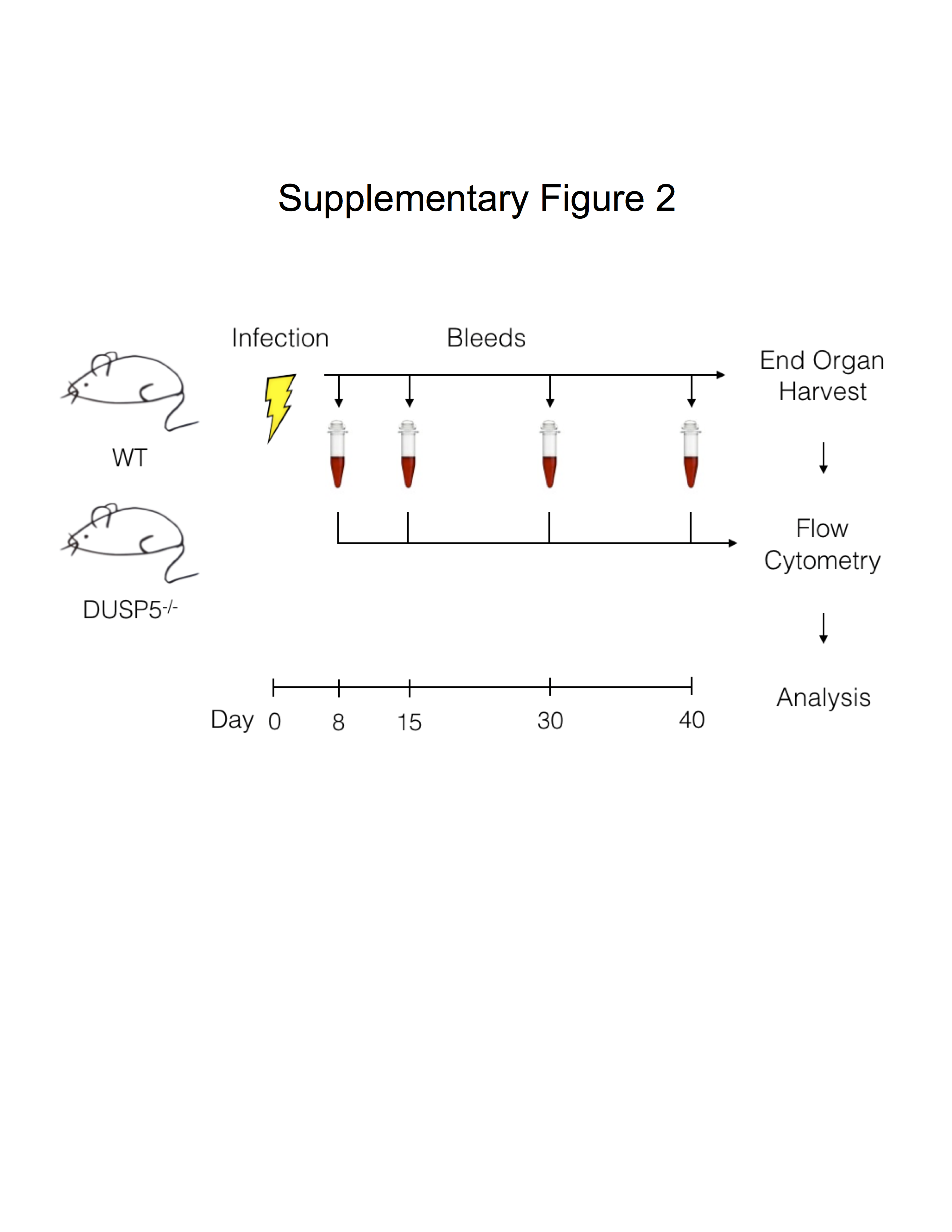

Supplement: S2 Fig — WT and Dusp5-/- mice are infected at day 0 with blood samples collected at days 8, 15, 30, and 40. At 40 days animals were sacrificed and organs were harvested for T cell population analysis via flow cytometry. (TIF) [file pone.0167246.s002.tif]

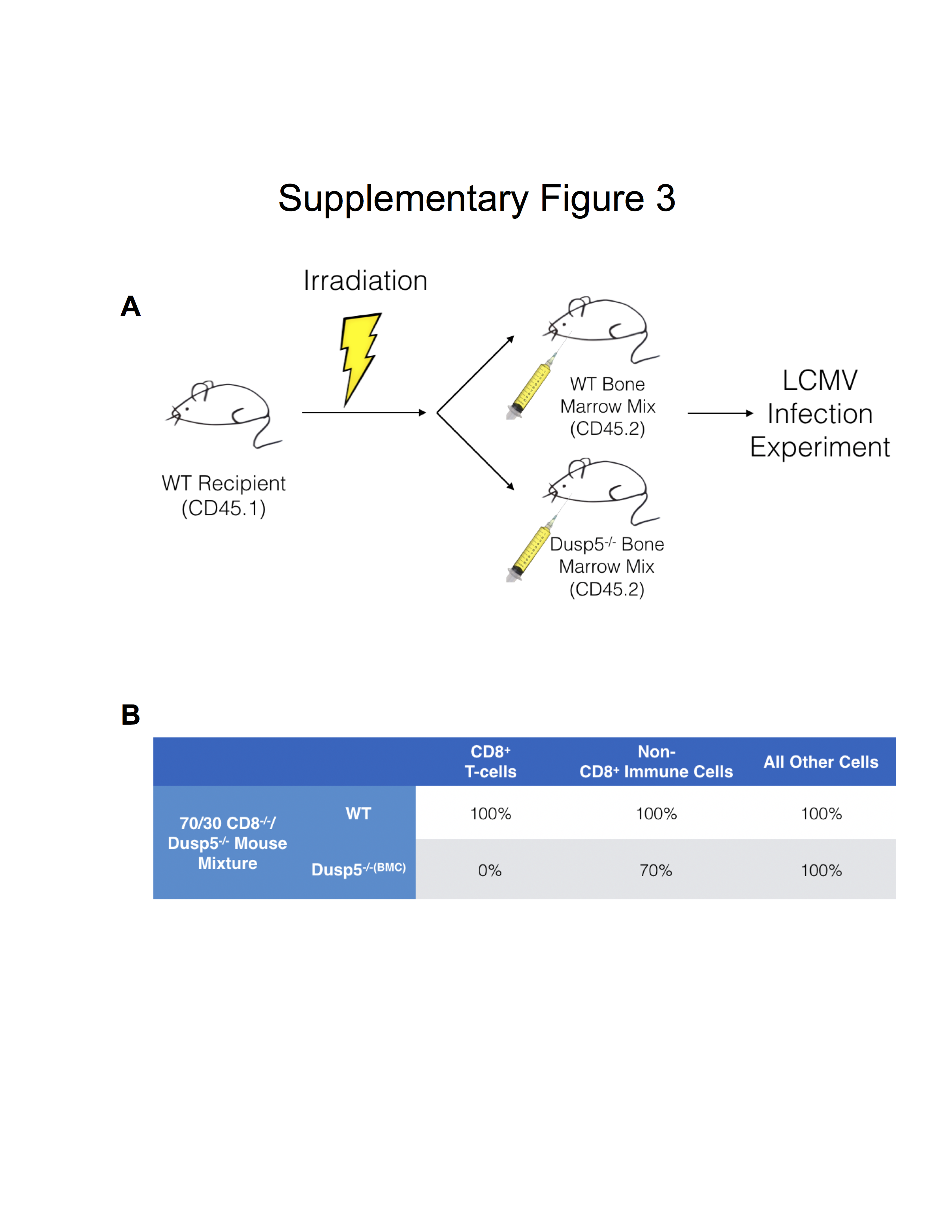

Supplement: S3 Fig — WT Ly5.1 (CD45.1) mice were lethally irradiated and subsequently injected with a mixture of Cd8-/- bone marrow and either Dusp WT or Dusp5-/- bone marrow in a ratio of 70:30. This was done to ensure that while Dusp5 was not expressed in CD8+ T cells, other lymphoid cell types would have Dusp5 expression. Once bone marrow was sufficiently reconstituted, mice participated in the LCMV infection model as described in S2 Fig. (TIF) [file pone.0167246.s003.tif]

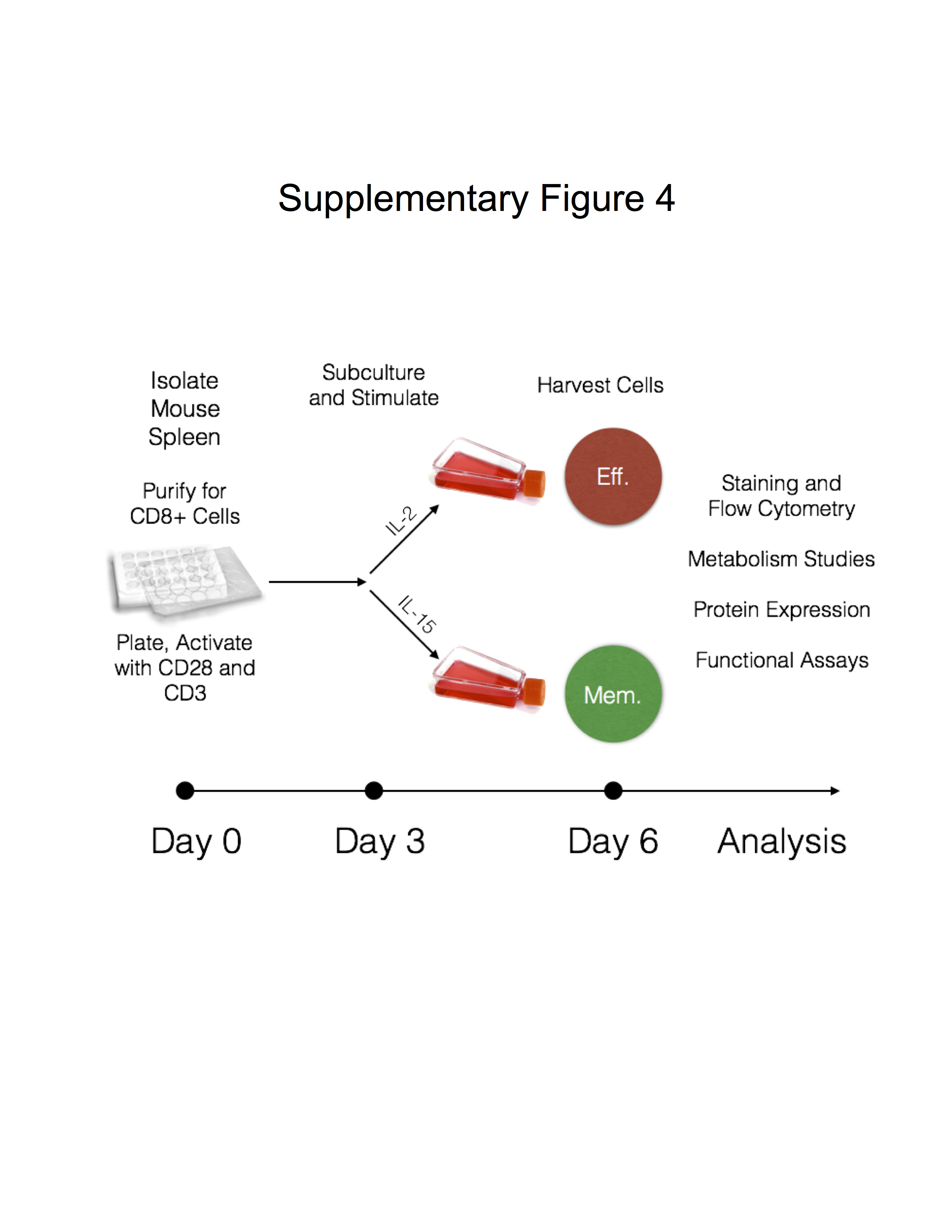

Supplement: S4 Fig — Spleen and lymph node were isolated from mice and reduced to single-cell suspension. These suspensions were purified for CD8+ CD44- naïve T cells and activated with anti-CD3 and anti-CD28 antibodies for three days. Cells were then sub-cultured into SLECs via IL-2 supplemented media or MPECs via IL-15 supplemented media. After 3 days of subculture, cells were collected for in vitro experiments. (TIF) [file pone.0167246.s004.tif]

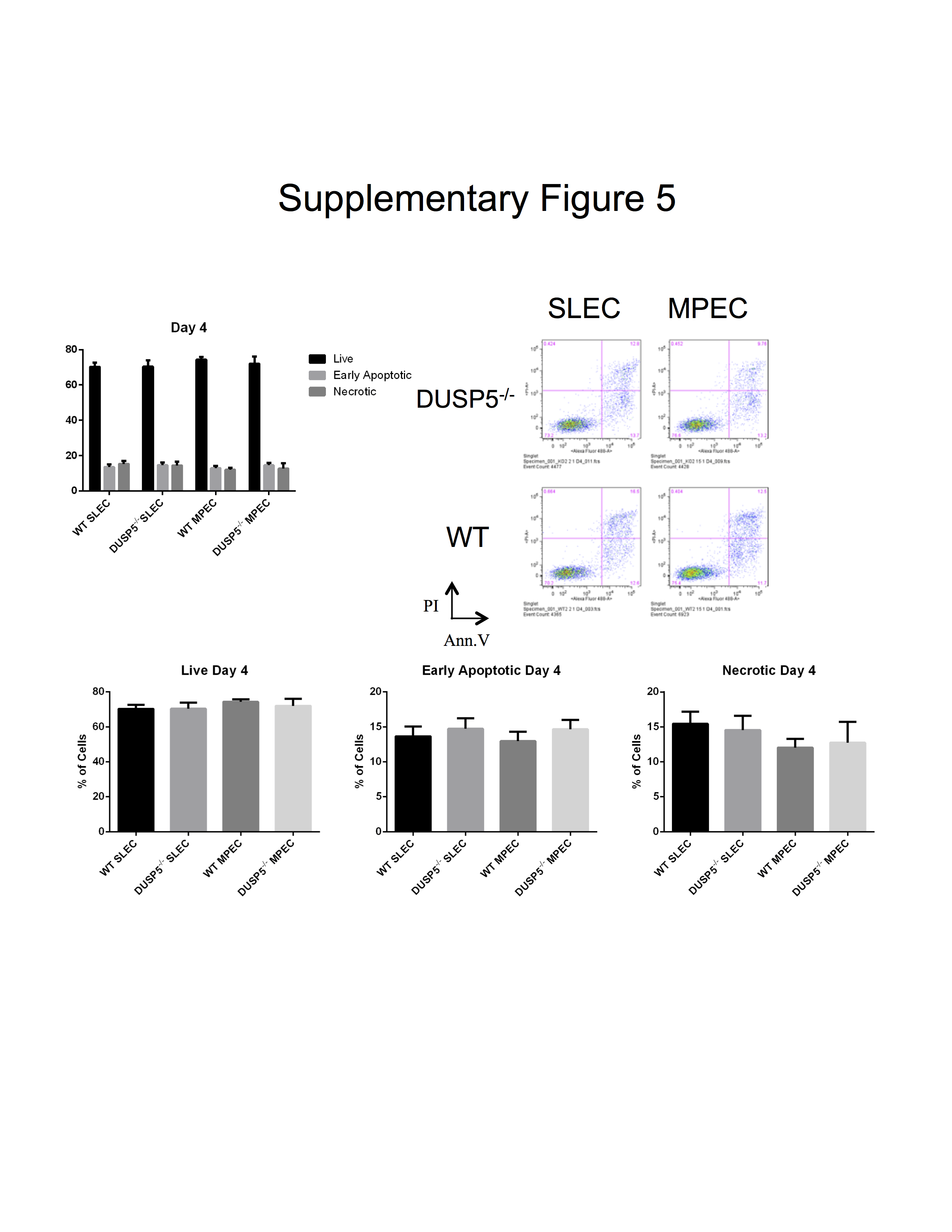

Supplement: S5 Fig — Neither SLEC nor MPEC cultured cells showed any differences between live, early apoptotic, or necrotic cells. Cell viability was decided using AnnexinV/Propidium Iodide staining and flow analysis. (TIF) [file pone.0167246.s005.tif]

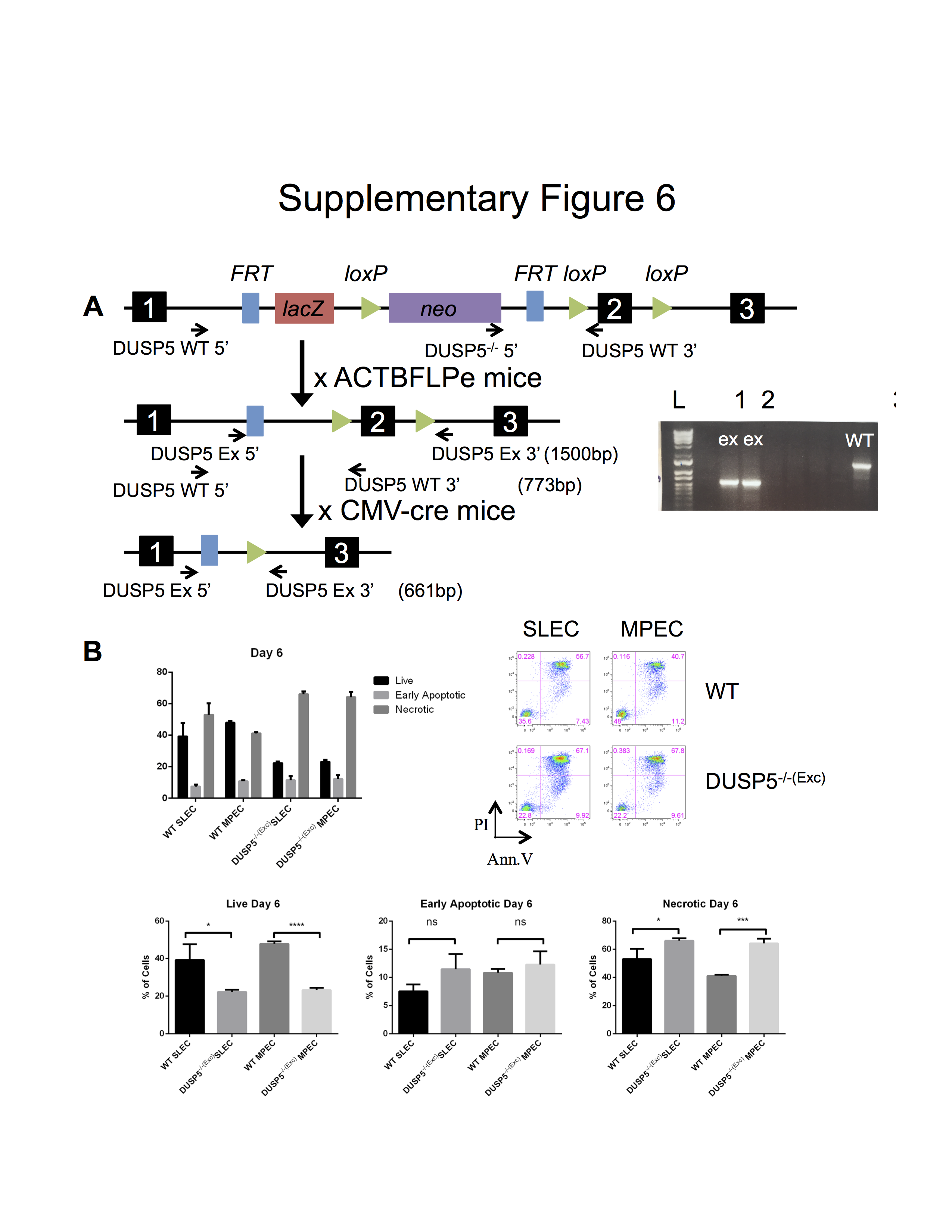

Supplement: S6 Fig — A: schematic of crossing strategies to first remove the lacZ/neo cassettes and, second, to remove the second exon of DUSP5 (this line then termed “Dusp5-/-(Exc)”. Lane description in gel image: Ladder (L), Dusp5-/-(exc) (1,2) and DUSP5WT/WT (3). WT alleles produced 1500bp PCR products while Dusp5-/-(exc) alleles produced 661bp PCR products. B: confirmation of phenotype. T cells from Dusp5-/-(Exc) mice were isolated and cultured as described above, with apoptosis data collected as also described. For each sample, n = 3, *: p<0.05, **: p<0.01 ***: p<0.005, ****p<0.001. (TIF) [file pone.0167246.s006.tif]

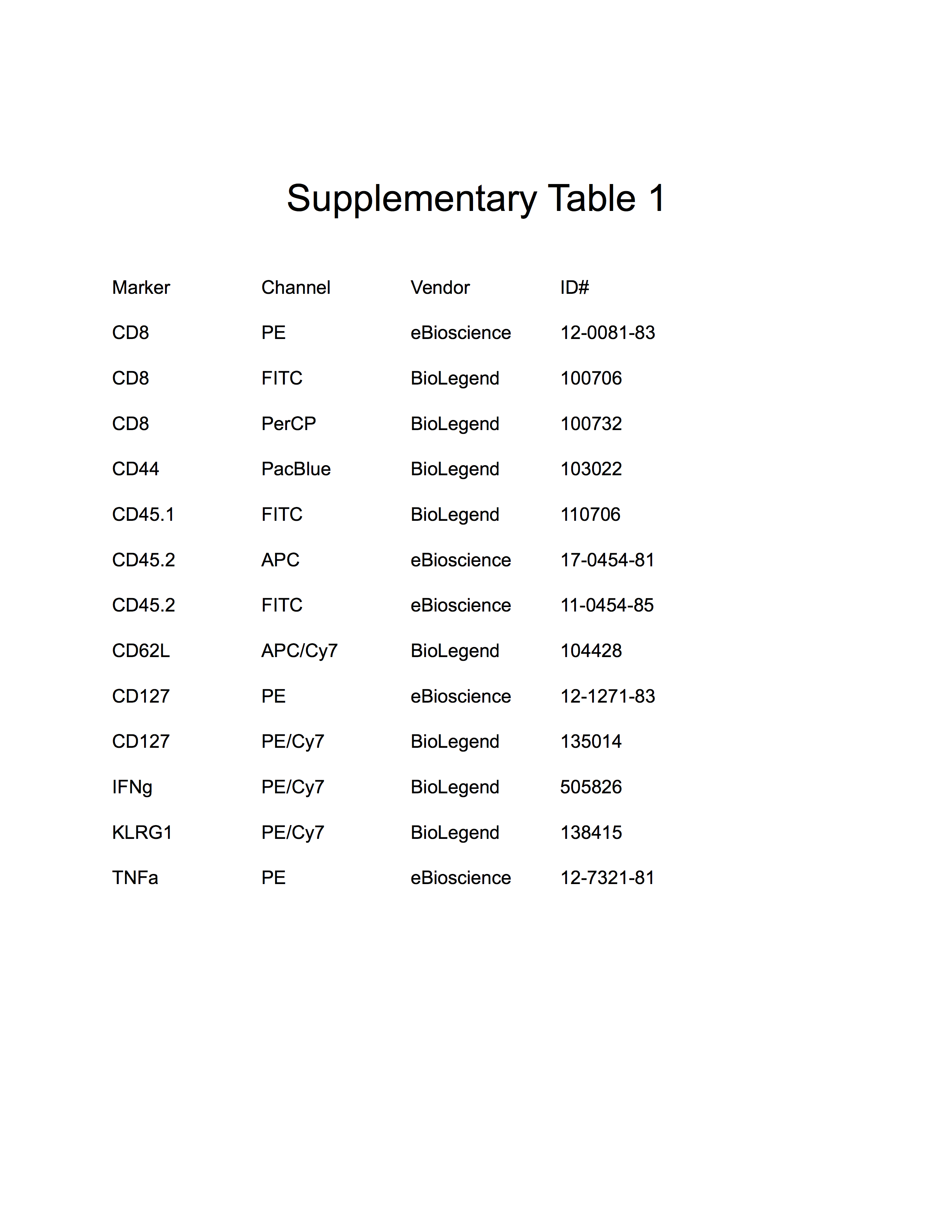

Supplement: S1 Table — (TIF) [file pone.0167246.s007.tif]
